# Modulation of Sensory Nerve Function by Insulin: Possible Relevance to Pain, Inflammation and Axon Growth

**DOI:** 10.3390/ijms21072507

**Published:** 2020-04-04

**Authors:** Bence András Lázár, Gábor Jancsó, Péter Sántha

**Affiliations:** 1 Department of Psychiatry, University of Szeged, H-6725 Szeged, Hungary; 2 Department of Physiology, University of Szeged, H-6720 Szeged, Hungary; gaborjancso@yahoo.co.uk (G.J.); santha.peter@med.u-szeged.hu (P.S.)

**Keywords:** insulin, insulin receptor, primary sensory neurons, capsaicin, TRPV1 receptor, pain, diabetes, pancreatitis, inflammation, neurite outgrowth, neuropathy

## Abstract

Insulin, besides its pivotal role in energy metabolism, may also modulate neuronal processes through acting on insulin receptors (InsRs) expressed by neurons of both the central and the peripheral nervous system. Recently, the distribution and functional significance of InsRs localized on a subset of multifunctional primary sensory neurons (PSNs) have been revealed. Systematic investigations into the cellular electrophysiology, neurochemistry and morphological traits of InsR-expressing PSNs indicated complex functional interactions among specific ion channels, proteins and neuropeptides localized in these neurons. Quantitative immunohistochemical studies have revealed disparate localization of the InsRs in somatic and visceral PSNs with a dominance of InsR-positive neurons innervating visceral organs. These findings suggested that visceral spinal PSNs involved in nociceptive and inflammatory processes are more prone to the modulatory effects of insulin than somatic PSNs. Co-localization of the InsR and transient receptor potential vanilloid 1 (TRPV1) receptor with vasoactive neuropeptides calcitonin gene-related peptide and substance P bears of crucial importance in the pathogenesis of inflammatory pathologies affecting visceral organs, such as the pancreas and the urinary bladder. Recent studies have also revealed significant novel aspects of the neurotrophic propensities of insulin with respect to axonal growth, development and regeneration.

## 1. Introduction

Insulin is a major hormone regulating the energy metabolism of the organism. Besides its endocrine effects, insulin may modulate distinct neural functions through insulin receptors (InsRs) expressed in neurons of the central and peripheral nervous system. Neuronal localizations of insulin and InsRs have been first described in the central nervous system mostly in areas involved in the regulation of energy balance and food intake [1]. Later studies disclosed widespread brain distribution of InsRs [1,2,3,4], and also that of insulin, which is of peripheral (plasma) rather than neuronal origin [5]. Available data indicate that most insulin is made in the pancreas, and there is no convincing evidence that insulin can be synthesized in the brain [5,6].

InsR, as a member of the receptor tyrosine kinase family, has a heterotetrameric structure with two extracellular α and two transmembrane glycoprotein β subunits which contain the tyrosine kinase domain. Insulin activates the InsR by binding to the α subunit of the receptor which induces the phosphorylation of tyrosine residues of intracellular proteins such as the insulin receptor substrate (IRS) proteins [7,8]. Phosphorylation of IRS proteins initiates the activation of phosphatidylinositol-3-kinase (PI3K) which, in turn, activates protein kinase B (Akt) and protein kinase C (PKC) cascades, and growth factor receptor bound protein 2/son of sevenless protein cascade which activates mitogen-activated protein kinase (MAPK) [7,9,10]. The Akt cascade is involved in the translocation of the glucose transporter 4 into the plasma membrane, regulation of glycogen, protein and lipid synthesis and glucose intake, while MAPK is related to the modulation of gene expression, proliferation and cell growth [11,12,13,14]. The tissue distributions of InsR and IRS proteins are different, suggesting their involvement in disparate physiological mechanisms and differing pathophysiological processes [7,15,16,17,18].

In recent years, electrophysiological, immunohistochemical and functional studies demonstrated the localization and functional significance of InsRs in primary sensory neurons (PSNs), in particular nociceptive PSNs expressing the transient receptor potential vanilloid type 1 (TRPV1) receptor [19,20,21,22,23,24,25]. The present review attempts to summarize experimental data on the diverse roles of insulin and InsRs in physiological and pathophysiological processes involving PSNs.

## 2. Subpopulation of Multifunctional Nociceptive PSNs Expressing TRPV1 Receptor

Nociceptive PSNs transmit impulses evoked by noxious mechanical, thermal and chemical stimuli [26]. Morphologically, nociceptive PSNs are mostly dark, type B sensory ganglion cells emitting thinly myelinated Aδ or unmyelinated C fibers [27,28,29,30,31,32,33,34]. It has been revealed that a group of nociceptive PSNs, if stimulated, release vasoactive agents to produce local vascular reactions, vasodilatation and plasma extravasation, collectively termed the neurogenic inflammatory response [35,36,37,38]. Nociceptive PSNs have been suggested to possess not only afferent functions by transmitting nociceptive impulses but also efferent/local regulatory functions through the release of vasoactive agents from their stimulated peripheral nerve endings [35,36,38]. It has also been demonstrated that nociceptive PSNs are sensitive to and can be selectively eliminated by capsaicin and comprise a morphologically, neurochemically and functionally distinct subpopulation of somatic and visceral PSNs [33,34,39]. Chemo-/capsaicin-sensitive PSNs have been shown to express the TRPV1 ion channel, the capsaicin receptor [40]. The TRPV1 receptor is generally regarded as a molecular integrator of nociceptive stimuli and can be activated by noxious heat (~43 °C), acidic pH, and exogenous (capsaicin) and endogenous vanilloids (e.g., anandamide) [41,42,43,44]. Furthermore, it has been revealed that various agents released upon tissue damage and inflammation, such histamine, prostaglandin E2, bradykinin and nerve growth factor (NGF) sensitize the TRPV1 receptor [44,45,46,47]. Capsaicin-sensitive PSNs contain different neuropeptides, such as substance P (SP), calcitonin gene-related peptide (CGRP) and somatostatin [48,49,50]. There is ample evidence for CGRP and SP mediating sensory neurogenic vasodilatation and sensory neurogenic plasma extravasation, respectively [51,52,53,54,55,56]. Besides playing a pivotal role in the initiation of vascular reactions, capsaicin-sensitive TRPV1 receptor expressing peptidergic PSNs are also involved in local regulation of smooth muscle contraction/relaxation, glandular secretion, immune reactions and other visceral functions [55,56,57,58,59]. Hence, neurons of this particular subpopulation of capsaicin-sensitive PSNs are multifunctional: they are involved not only in the transmission of nociceptive impulses, but also in local regulatory functions of the innervated organs. Therefore, agents interfering with TRPV1 receptor function may modulate both the afferent and local regulatory functions of PSNs.

Immunohistochemical studies have revealed that capsaicin-sensitive PSNs can be divided into two major categories: peptidergic and non-peptidergic neurons. These subpopulations of PSNs have different trophic factor sensitivities and neurochemical characteristics. TRPV1 receptor-expressing peptidergic PSNs contain CGRP, SP, vasoactive intestinal polypeptide, galanin and neurokinin A [48,50,60,61]. Non-peptidergic nociceptive PSNs express fluoride-resistant acid phosphatase, equivalent to thiamine monophosphatase [62,63,64,65,66,67], an enzyme which has been recently identified as prostatic acid phosphatase [68]. Furthermore, it has been revealed that non-peptidergic neurons bind the isolectin B4 (IB4) from *Bandeiraea simplicifolia* [63,69]. Besides their different neurochemical characteristics, peptidergic and non-peptidergic PNSs show disparate neurotrophin sensitivities as well. Although, during the early postnatal period, peptidergic and non-peptidergic PSNs are responsive to NGF and express the tropomyosin kinase A (TrkA), later non-peptidergic neurons lose their NGF-sensitivity by the down-regulation of TrkA, and they become sensitive to glial cell line-derived neurotrophic factor and express the Ret [70,71,72]. Although, neurochemical characteristics and neurotrophins sensitivities of peptidergic and non-peptidergic nociceptive neurons show differences, up to 60% of neurons of these subcategories of PSNs express the archetypal nociceptive ion channel, the TRPV1 receptor [22,23,40,73].

Characterization of the chemical phenotypes of PSNs have revealed the sensitivity to insulin and the localization of InsRs in a sizeable subpopulation of neurons amounting to about 60 per cent of spinal PSNs of unidentified target identity [19,22,23] Further, it has also been demonstrated that insulin and insulin-like growth factor 1 (IGF-1) can also sensitize the TRPV1 receptor [19]. These findings suggested a possible interplay among insulin, InsR and TRPV1 receptor in PSNs under pathophysiological conditions.

## 3. Neurochemical Characterization of Somatic and Visceral PSNs Expressing the InsR

Early light and electron microscopic studies demonstrated the presence of InsR immunoreactivity in a population of small dorsal root ganglion (DRG) neurons and in Rexed’s laminae V and X of the spinal cord [74]. At the ultrastructural level, InsR is localized on the axolemma, the Schwann cell loops and the nodal microvilli of myelinated peripheral nerves [75]. Studies on cultured PSNs supported these original findings by showing that about 50% of the neurons expressed the InsR and up to 30% of the neurons showed colocalization of the TRPV1 receptor and the InsR [19]. Moreover, it has been demonstrated that about half of the TRPV1 receptor-immunoreactive PSN neurons exhibited the InsR. The colocalization of the InsR and neuropeptides has also been revealed: about 20% of InsR-expressing mouse DRG neurons showed CGRP and/or IB4-immunoreactivity [22]. In addition, it has also been demonstrated that there is no difference in the immunoreactivities of these markers between wild-type and TRPV1 receptor knock out mice [22].

Later, the expression pattern of InsR and the co-localization the InsR with the TRPV1 receptor, CGRP and IB4 have also been revealed in cultured rat DRG neurons. It has been demonstrated that about half of the DRG neurons showed InsR-immunopositivity and about 60%, 50% and 30% of InsR-expressing sensory neurons exhibited the TRPV1 receptor, CGRP and the IB4-binding glycoprotein, respectively [23]. Furthermore, it has been revealed that about 60% and 50% of TRPV1 receptor- and CGRP- or IB4-immunopositive cultured DRG neurons exhibited InsR-immunoreactivity [23].

The neurochemical phenotypes of InsR-expressing PSNs innervating different organs have also been revealed. It has been demonstrated that about one-quarter of somatic (cutaneous and muscle) and about half of visceral (urinary bladder and pancreatic) PSNs express the InsR [24]. In addition, it has been demonstrated that the differences between the two subpopulations (somatic and visceral) were significant [24]. However, about 60% of InsR-expressing PSNs innervating the dorsal hind paw skin, the gastrocnemius muscle, the urinary bladder and the pancreas showed TRPV1 receptor immunoreactivity [24]. These observations suggest that the majority of InsR-expressing PSNs are nociceptive in nature.

The phenotypes of pancreatic spinal and vagal PSNs have been analyzed in more detail. It has been demonstrated that about 50% of DRG and nodose ganglia (NG) neurons innervating the rat pancreas express the InsR [25]. It has also been shown that up to 50% of InsR-expressing pancreatic spinal and vagal PSNs exhibit TRPV1 receptor immunoreactivity [25]. Further, about 30% of InsR-expressing pancreatic spinal and vagal sensory neurons contain SP. CGRP is contained in about 60% and 20% of spinal and vagal PSNs, respectively [25]. Moreover, it has been revealed that up to 30% of TRPV1 receptor-expressing pancreatic spinal and vagal PSNs exhibit InsR immunoreactivity [25]. In addition, up to one-quarter of CGRP or SP containing pancreatic spinal and vagal sensory neurons express the InsR as well [25].

In summary, these quantitative immunohistochemical studies have revealed the disparate localization of InsRs in somatic and visceral PSNs with dominance of neurons innervating visceral organs. This suggests that visceral PSNs involved in nociceptive and inflammatory processes may be more prone to the modulatory effects of insulin than somatic PSNs. Additionally, these studies have demonstrated that majority of InsR-expressing PSNs also display TRPV1 receptor immunoreactivity. Importantly, these observations have revealed that the pancreas is innervated by a meshwork of InsR-expressing spinal and vagal PSNs which contain sensory neuropeptides and express the TRPV1 receptor. These morphometric data implicate that an interaction among insulin, InsR, TRPV1 receptor and sensory neuropeptides may play an important role in the development of pancreatic pathologies.

## 4. Effects of Insulin on TRPV1 Receptor-Expressing Nociceptive Neurons

Studies on cultured adult rat DRG neurons have revealed that insulin can sensitize the TRPV1 receptor toward the effect of the selective agonist capsaicin. It has been demonstrated that administration of insulin enhances the accumulation of cobalt ions in capsaicin-stimulated neurons [19]. The cobalt accumulation technique relies on the histochemical visualization of cobalt ions selectively taken up by neurons expressing the non-selective cation channel TRPV1 upon stimulation with agonists, such as capsaicin. Densitometric analysis of the precipitated cobalt ions is a reliable tool to estimate the proportion and the reactivity of individual capsaicin-sensitive PSNs in culture [76,77]. An advantage of this method is that cobalt ions enter the cells exclusively through the activated TRPV1 channels but not through voltage sensitive Ca^2+^ or Na^+^ channels; hence, cobalt accumulation specifically indicates changes in the conductance of plasma membrane TRPV1 channels upon agonist-induced activation [78]. The sensitizing effect of insulin could be reduced by inhibitors of tyrosine-kinase, phospholipase C and PKC [19]. Furthermore, at the highest concentration applied (10 µM), insulin induced cobalt accumulation *per se* indicating the activation of the TRPV1 receptor. The insulin-induced cobalt accumulation was reduced by administration of both competitive and non-competitive antagonists of the TRPV1 receptor, capsazepine and ruthenium red, respectively, further suggesting the involvement of TRPV1 receptor in the action of insulin. Whole-cell voltage clamp experiments disclosed that acute administration of insulin induced inward currents in subsets of cultured PSNs of both the rat (18%) and the mouse (9%) in the concentration range of 300 nM-10 µM [22,79]. All neurons responding to insulin were also sensitive to capsaicin, and ruthenium red at concentrations sufficient to block capsaicin-induced currents also abolished the insulin-induced currents. Importantly, in cultures prepared from DRGs taken from TRPV1 receptor knock out mice, both the number of neurons showing activation of inward current following insulin application and the maximal current intensity evoked by insulin were significantly lower compared to data obtained from wild type PSNs [79]. These results strongly suggest, that besides its long-term neurotrophic effects, insulin may modulate the chemo- and thermosensitivity of nociceptive PSNs co-expressing the TRPV1 receptor and the InsR. Insulin-mediated sensitization of the TRPV1 receptor currents induced by chemical or thermal stimulations were also demonstrated in heterologous expression systems and isolated embryonic DRG neurons [20]. The sensitizing effect of insulin is mediated in part by rapid translocation and insertion of TRPV1 channels into the plasma membrane, an effective mechanism to increase the sensitivity of sensory neurons toward noxious stimulation.

Furthermore, increased expression of TRPV1 receptor protein has been demonstrated following long-term administration of IGF-1 and insulin in TRPV1 receptor-expressing neuroblastoma cells. In addition, it has been disclosed that expression of TRPV1 receptor protein is decreased by inhibiting the MAPK and PI3K cascades. These findings suggested a crucial role of MAPK and PI3K signaling pathways in the regulation of the TRPV1 receptor by IGF-1 and insulin [21]. In accord with these observations, the involvement of PI3K and PKC cascades has also been demonstrated in the regulation of TRPV1 receptor mediated membrane currents, which are enhanced by IGF-1 and insulin [20].

Similarly, acute administration of NGF has been shown to induce translocation of intracellular TRPV1 receptors into the plasma membrane, underlying its sensitizing effect on the TRPV1 channel [80]. It is worth to mention, that IGF-1, widely expressed and secreted in different tissues, can also sensitize [20] and activate [79] cultured nociceptive neurons. It has also been shown that long-term application of insulin modifies the expression of the TRPV1 receptor protein in neuroblastoma cells by regulating the PI3K and MAPK signaling [21]. Additionally, in a recent publication it has been demonstrated, that selective chemodenervation evoked by neonatal capsaicin treatment resulted in changes in myocardial miRNA expression targeting among others the expression network of IGF-1 [81].

Studies utilizing calcium imaging demonstrated the activation of a subset of mouse NG neurons by insulin in a dose-dependent manner [82]. All neurons which were sensitive to insulin were also sensitive to capsaicin and expressed the TRPV1 receptor. By using selective antagonists, the involvement of L-type (verapamil) and N-type (ω-conotoxin) voltage-sensitive Ca-channels in the activation of NG neurons by insulin has also been revealed. These findings suggested that apart from TRPV1, other (Ca) channels are also involved in the direct activation of sensory neurons by insulin. To demonstrate the direct involvement of InsR signaling in the insulin-mediated effect, a genetically modified mice strain was used. Genetic deletion of the insulin receptor substrate type 2 protein (IRS2) completely inhibited the insulin-induced depolarization and calcium influx in cultured NG neurons. Similarly, blocking of PI3K, one of the main intracellular messengers of the insulin signal resulted in a marked reduction in the incidence of calcium responses to insulin. These data indicated that the signal cascade InsR-IRS2-PI3K plays an essential role in the activation of NG neurons by insulin, although other pathways may also be involved [82]. Importantly, it has been shown that more than 50% of neurons responsive to insulin expressed the neuropeptide cocaine and amphetamine regulated transcript (CART). Since CART exerts powerful anorexigenic actions in both the hypothalamus and the nucleus of the solitary tract, an involvement of insulin-sensitive pancreatic vagal afferents in the regulation of food intake, satiety and metabolism has been proposed [82].

## 5. Contribution of TRPV1 Receptor- and InsR-Expressing PSNs to Pancreatic and Urinary Bladder Pathologies

It has been demonstrated that a TRPV1 receptor antagonist, capsazepine decreases the expression level of neurokinin-1 receptor, the receptor of SP, the pancreatic enzymes associated with pancreatitis, and the severity of histological changes in caerulein-induced experimental pancreatitis in mice. These findings suggested the importance of the TRPV1 receptor expressing SP-immunopositive fibers in pancreatic pathologies [83]. Furthermore, it has been revealed that neonatal capsaicin treatment causes a significant decrease of inflammatory markers and histological changes of the pancreas in experimental acute pancreatitis [84]. Other studies have demonstrated that the expression of the TRPV1 receptor is increased in DRG neurons during the development of chronic pancreatitis [85]. Moreover, it has been revealed that administration of TRPV1 receptor antagonists reduce the severity of acute pancreatitis [86]. These findings have provided evidence for a crucial role of the TRPV1 receptor in the mediation of pathologies of the exocrine pancreas.

The TRPV1 receptor is also expressed in the endocrine pancreas, and it has also been demonstrated that activation of the TRPV1 receptor can modulate the glucose-induced insulin secretion of pancreatic islet β cells [87,88]. The direct effect of sensory neuropeptides on pancreatic islet β cells has also been revealed [89,90,91,92]. Previous studies have demonstrated that pancreatic islets are innervated by TRPV1 receptor expressing sensory nerves which can be eliminated by neonatal capsaicin treatment [91]. Interestingly, in an experimental model of type 1 diabetes mellitus (non-obese diabetic (NOD) mice) neonatal capsaicin treatment delayed the onset of diabetes and reduced its incidence compared to control NOD mice. It has also been revealed that neonatal capsaicin treatment reduced the accumulation of inflammatory cells including T-cells throughout the islets. Importantly, it has also been revealed that TRPV1 receptor protein expression is decreased in control NOD mice [91]. Furthermore, it has been demonstrated that pancreatic PSNs which contain sensory neuropeptides have a protective role against the development of diabetes mellitus [91,93]. It has also been shown that systemic administration of capsaicin prevents the development of decreased glucose tolerance in an animal model (Zucker diabetic rat) of type 2 diabetes mellitus [94]. Importantly, a novel study has demonstrated that administration of a TRPV1 receptor antagonist improved the glucose-stimulated insulin secretion and glucose tolerance in Zucker diabetic rats [95]. These studies suggested the importance of sensory neurons expressing the TRPV1 receptor in the mediation of pancreatic pathologies. Recently, loss of InsRs expressed by pancreatic sensory nerves has been shown to result in elevated insulin levels and reduced glucose tolerance in sensory neuron insulin receptor knock out (SNIRKO) mice [96].

Recent work has indicated that the TRPV1 receptor may play an important modulatory role in glucose-induced insulin secretion as well. It has been demonstrated that TRPV1 receptor knock out mice show significantly higher blood glucose levels, and a higher decrease in glucose induced insulin secretion compared with wild type mice [97]. Additionally, it has been revealed that capsaicin can increase pancreatic insulin secretion of wild-type, but not of TRPV1 receptor knock out animals [97]. Furthermore, it has been shown that high glucose stimulation causes significantly higher pancreatic CGRP and SP release in wild-type compared to knock out mice. Interestingly, administration of TRPV1 receptor, CGRP or SP antagonists reduced insulin secretion in wild-type, but not in knock out mice [97]. This study supported previous findings by showing an important role of TRPV1 receptor and sensory neuropeptides in the regulation of glucose-induced insulin secretion of the pancreas. Another study demonstrated that intragastric administration of allyl isothiocyanate, which is a potent agonist of the transient receptor potential ankyrin type 1 (TRPA1) receptor and activates the TRPV1 receptor as well, elevates plasma insulin levels in mice by directly stimulating insulin secretion from the pancreas [98]. Moreover, it has been revealed that in TRPV1 receptor knock out mice, the allyl isothiocyanate-induced insulin secretion was lower compared to TRPV1 receptor wild-type animals [98].

Taken together, these studies have demonstrated that insulin may potentiate the release of inflammatory neuropeptides by sensory nerves innervating the islet apparatus and the exocrine pancreas through direct and/or indirect modulation of TRPV1 receptors [82,86,91,94,99,100,101,102]. Further, neuropeptides contained in sensory nerves, such as SP and CGRP may modulate inflammatory processes of both the endocrine and exocrine pancreas. Quantitative immunohistochemical findings supported these findings by showing that the majority of pancreatic vagal and spinal DRG neurons express the TRPV1 receptor, the InsR and sensory neuropeptides [25].

The role of capsaicin-sensitive sensory nerves in the function and pathologies of the urinary bladder is well established. Functional morphological studies have shown that a high proportion of sensory nerves which innervate the urinary bladder are mostly unmyelinated and capsaicin-sensitive [59,103]. Activation of these TRPV1 receptor expressing nerves has been shown to produce contraction of the bladder smooth muscle and reflex micturition. Depletion of sensory neuropeptides by systemic pretreatment with capsaicin greatly inhibited or even abolished reflex micturition in rats [104]. Capsaicin-sensitive nerves have been implicated in human bladder pathologies such as pain associated with overactive bladder [105,106]. Intravesical capsaicin has been shown to ameliorate symptoms of detrusor hyperreflexia and is in clinical use to treat this condition [107]. A decrease in TRPV1 receptor agonist-induced urinary bladder contraction has been shown in streptozotocin-induced experimental diabetes [108]. This may be explained by a loss of sensory nerves, impaired axonal transport or expression of the TRPV1 receptor [108]. Importantly, changes in TRPV1 receptor gene expression may also contribute to a decrease of TRPV1 receptor agonist-induced smooth muscle contraction since, in neuroblastoma cells, TRPV1 receptor gene transcription has been shown to be insulin-dependent [21]. Insulin has also been shown to restore the TRPV1 receptor-mediated vasodilatatory response of dura mater arterioles in streptozotocin diabetic rats [109]. Activation of capsaicin-sensitive afferent nerves, through the release of pro-inflammatory peptides SP and CGRP, has also been shown to contribute to urinary bladder inflammation induced by cyclophosphamide [110]. These findings suggest interactions among TRPV1 receptors, insulin and the InsRs which may significantly modulate functions of different organs, including urinary bladder smooth muscle contractility, inflammation and nociception. It is also to be noted, however, that several studies have demonstrated that insulin inhibits general inflammatory processes as well. It has been revealed that insulin suppresses the expression of inflammatory chemokines such as nuclear factor-kappaB and monocyte chemoattractant protein-1 in vitro [111]. Additionally, it has been shown that in vitro treatment with insulin decreases tumor necrosis factor and interleukin 6 expression by regulating the PI3K-Akt cascade [112,113].

## 6. Role of TRPV1 Receptor, InsR and Insulin in the Development of Complications Associated with Diabetes Mellitus

Over the past few years, many reports have indicated that nociceptive sensory neurons are involved in the development of diabetic peripheral neuropathy and diabetic visceral hypo- or hypersensitivity [91,99,114,115,116,117,118,119,120]. Furthermore, there is growing evidence that supports an important role of TRPV1 receptor-mediated neuroinflammation through the release of sensory neuropeptides in the development of diabetic peripheral neuropathy [121].

Recent work using immunohistochemistry and western blot analysis has revealed that expression of TRPV1 receptor and CGRP decreased in spinal cord dorsal horn of in rats with streptozotocin (STZ) diabetes. It has also been demonstrated that expression of these markers were also reduced in peripheral nerves [122]. In addition, it has been shown that ropivacaine, which is a commonly used anesthetic in the treatment of peripheral neuropathy, has a protective effect against the development of diabetic peripheral neuropathy by regulating TRPV1 receptor mediated CGRP release [122]. In agreement with this study, another report has demonstrated that TRPV1 receptor antagonists such as capsazepine prevent hydrogen sulfide-induced hyperalgesia in diabetic rats [123]. This work suggested that TRPV1 receptor blockers could be a therapeutic target for the treatment of diabetic peripheral neuropathy [123].

Additionally, in the past decade, several lines of evidence have suggested that insulin has a direct role on PSNs and insulin signaling may be involved in the development of diabetic peripheral neuropathy [124,125,126,127]. Regarding the potent neurotrophic effect of insulin, recent studies have indicated that beside glucose toxicity, insulinopenia and hyperinsulinemia may be important pathogenic components of diabetic peripheral neuropathy and visceral hypo- and hypersensitivity [25,126,127,128,129].

It has also been revealed that insulin infusion causes a decrease in CGRP-mediated sensory neurogenic vasodilatation in rats [116]. Furthermore, it has been demonstrated that chronic hyperinsulinemia induced by fructose (fructose-drinking rats, FDR) also inhibits the vasodilatation mediated by peptidergic sensory fibers [117]. Interestingly, it has been revealed that DRGs of FDRs showed a decreased CGRP content and reduced density of CGRP-immunoreactive fibers in mesenteric arteries [119]. Moreover, in the last decade, several reports has demonstrated that mitochondrial dysfunction of DRG neurons is involved in the development of diabetes mellitus [130,131,132]. It has also been proposed that loss of insulin signaling is involved in the development of mitochondrial changes of DRGs [126,133,134]. Interestingly, a recent study has revealed that insulin promotes mitochondrial gene expression of cultured DRG neurons derived from control and STZ- diabetic rats [135]. Additionally, it has also been demonstrated that insulin treatment enhanced thermal sensitivity and increased dermal nerve density in diabetic animals [135]. It has also been shown that chronic administration of fructose, which induces insulin resistance, and neuropathic pain, reduced InsR protein expression in DRGs and sciatic nerve [123]. However, it has also been revealed that SNIRKO mice do not show any physiological, morphological or behavioral signs of diabetic peripheral neuropathy [96].

In summary, these studies suggested a significant role of the TRPV1 receptor in the development of diabetic peripheral neuropathy and visceral hypo- or hypersensitivity. Although some of these experiments suggested that impairments in neuronal insulin signaling may contribute to the development of diabetic neuropathy, the role of interactions among insulin, InsRs and the TRPV1 receptors in these processes have not yet been evaluated in detail.

## 7. Role of Insulin and InsRs in Neurite Outgrowth and Regeneration of PSNs

Early studies have revealed that insulin has a potent neurite outgrowth-promoting effect on cultured adult rat sensory and sympathetic neurons [136]. Further, it has also been demonstrated that insulin promotes the neurite outgrowth of human neuroblastoma cells as well [137]. Following these observations, several studies have attempted to reveal the exact molecular mechanisms of insulin’s neurite outgrowth-promoting effects on sensory neurons.

Recent observations confirmed and extended the findings of previous reports on the neurite growth promoting effect of insulin. It has been revealed that insulin modulated neurite outgrowth in a concentration dependent manner. In cultured DRG neurons insulin administered at low (nM) concentrations elicited a growth promoting effect, whereas at micromolar concentrations an inhibition of axon growth was observed [138]. Importantly, in cultured DRG neurons, a significantly enhanced neurite growth promoting effect of insulin was observed in neurons which express the InsR, as compared to neurons which do not [23]. It has also been shown that insulin promotes axon growth by stabilization of tubulin mRNA levels [139].

The exact molecular background of insulin’s neurite outgrowth promoting effect has not yet been evaluated in detail. However, in the light of previous observations, it could be hypothesized that the interaction between insulin and the InsR by activating the PI3-Akt pathway [140,141], that is directly related to axonal growth, has a significant effect on neurite outgrowth of DRG neurons. Recent work has also shown that administration of high (µM concentrations of insulin, through the desensitization of the InsRs, render DRG neurons less sensitive to neurite outgrowth-promoting actions of insulin by inhibiting the PI3K-Akt pathway [141]. Furthermore, it has been revealed that increase of GSK-3b levels may contribute to the decreased sensitivity of DRG neurons to insulin [141,142].

Although, these studies suggested a crucial role of the InsR in neurite growth, it has also been demonstrated that the InsR forms a molecular complex with the TrkA in PC12 cells which show similarities with nociceptive sensory neurons [143]. Further, it has been demonstrated that TrkA is involved in insulin signaling by regulating the MAPK or Akt cascades [143]. TrkA, as a transmembrane receptor tyrosine kinase for NGF, is involved in neuronal survival and differentiation of PSNs (Silos-Santiago, 1995, Molliver). Thus, it may be assumed that the InsR and TrkA are involved in the mediation of the neurite outgrowth-promoting effect of insulin on nociceptive PSNs. This is supported by recent findings showing a preferential neurite outgrowth promoting effect of insulin on InsR-immunoreactive peptidergic subpopulations of cultured adult rat PSNs [23]. Available experimental evidence indicates that ganglioside GM1 is significantly implicated in the mediation of the neurotrophic effect of NGF in primary sensory neurons [144]. TRPV1 receptors are associated with and regulated by GM1, an important constituent of membrane lipid rafts [145,146,147]. Since InsRs are recruited upon ligand binding to membrane lipid microdomains [148], an interaction of the InsR with the TRPV1 receptor at the lipid raft domains of the plasma membrane cannot be excluded.

Importantly, in vivo studies have revealed that intrathecal [149] or systemic [150] administration of insulin stimulated the regeneration of injured peripheral nerves. IGF-I has also been shown to promote regeneration of sciatic nerve afferent fibers after local application to relating dorsal root ganglia or directly onto the injured nerve. Importantly, application of antibodies against IGF-I resulted in a significant reduction of axonal regeneration, suggesting a role for endogenous IGF-I in nerve regeneration [151]. In addition, local administration of IGF-II has been shown to enhance regeneration of motor axons following a crush injury. In contrast, spontaneous regeneration was inhibited by local application of an anti-IGF antiserum to the injured nerve [152]. Recent findings suggest that the neurotrophic effects of insulin and IGF-1, similarly to NGF, may be mediated through activation of TrkA [143]. TrkA is also involved in the development of the neurochemical phenotypes of nociceptive PSNs and, importantly, the expression of the TRPV1 receptor is regulated by NGF [77,153]. Since TrkA seems to be a common target of both insulin and NGF, a possible role of insulin and InsRs in the phenotypic regulation of nociceptive neurons cannot be excluded.

In vivo experiments have also indicated an increased insulin sensitivity of peptidergic PSNs. Thus, it has been revealed that administration of intrathecal insulin increased the expression of CGRP in regenerating nerve fibers and DRG neurons after nerve injury [149]. Additionally, it has also been demonstrated that local insulin treatment promoted the regeneration of myelinated fibers and small-fiber sprouting in streptozotocin diabetic rats [154]. Recent quantitative immunohistochemical data demonstrated that CGRP-immunoreactive PSNs displayed increased neurite outgrowth, axonal branching and neurite length as compared to CGRP-negative neurons. It has also been shown in these experiments that insulin did not increase neurite elongation of non-peptidergic PSNs [23]. Interestingly, streptozotocin, an agent used to induce experimental diabetes mellitus, has a direct effect on PSNs by increasing the expression of the TRPV1 receptor and the TRPV1 receptor-mediated CGRP-release [155].

Several reports have demonstrated that the density of CGRP-expressing perivascular sensory fibers and the CGRP content of DRGs decreased in mice with experimental insulin resistance [116,117,118,119]. In particular, these reports have also revealed that these fibers respond poorly to the trophic actions of exogenous NGF as compared to controls [118,119]. Similarly, capsaicin-induced release of CGRP and the density of CGRP and TRPV1 receptor expressing sensory fibers were markedly decreased in the dura mater of diabetic rats as compared to controls [109]. Hyperinsulinemia has been shown to induce insulin resistance of DRG neurons through hyperphosphorylation of the InsR by an Akt-mediated pathway, and the increase in the expression of GSK-3-β [156]. It has also been demonstrated that PSNs develop resistance to the trophic actions of insulin by the downregulation of InsR mRNA [141].

## 8. Conclusion and Perspectives

Besides its fundamental significance in energy metabolism, a significant role of insulin in modulation of neural functions has emerged. InsRs are expressed mostly on nociceptive TRPV1 receptor expressing PSNs innervating somatic and visceral organs with predominance in the latter. InsRs are involved in mechanisms of organ pathologies, in particular inflammatory processes of both the exocrine and endocrine pancreas. Experimental evidence indicates a pivotal role of TRPV1 receptor expressing PSNs in the pathomechanism of acute pancreatitis. Pancreatic TRPV1 receptor- and InsR-expressing peptidergic afferent nerves are also implicated in the mechanism of insulitis and islet dysfunction contributing to the development of type 1 diabetes mellitus.

**Figure 1:**
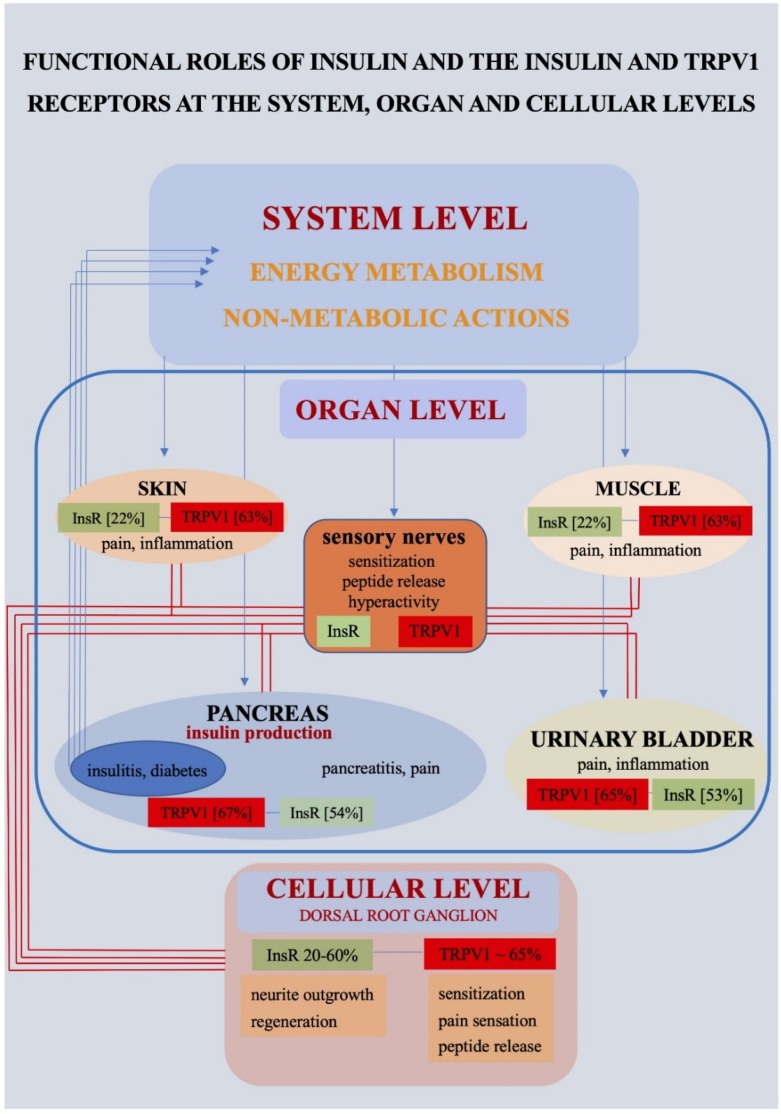
Insulin acts as a neuroactive substance by modulating the insulin (InsR) and transient receptor potential vanilloid type 1 (TRPV1) receptors at system, organ and cellular levels. The involvement of the InsR and the TRPV1 receptor in some physiological and pathophysiological processes is indicated. Percent values show proportions of dorsal root ganglion neurons expressing the InsR and the TRPV1 receptor innervating the organ.

Figure 1 summarizes the mostly interrelated functional roles of insulin and the InsR and the TRPV1 receptor at system, organ and cellular levels. Although the significance of modulation of TRPV1 receptor function in the therapeutic management of pain, inflammatory states and neurogenic dysfunction, such as overactive bladder is now well established [157], further studies are warranted to exploit the possible therapeutic value of the observations summarized in this review. InsRs expressed on PSNs play a significant role in the mechanisms of neurite outgrowth of cultured PSNs and axonal regeneration in vivo. The exploration of the possible significance of these findings in relation to the mechanism and therapy of neuropathic pathologies associated with diabetes mellitus awaits future studies.

## Abbreviations

Akt	protein kinase B
CGRP	calcitonin gene-related peptide
DRG	dorsal root ganglion
FDR	fructose-drinking rats
GSK-3-β	glycogen synthase kinase-3β
IB4	*Bandeiraea simplicifolia* isolectin B4
IGF-1	insulin-like growth factor 1
InsR	insulin receptor
IRS	insulin receptor substrate
MAPK	mitogen-activated protein kinase
NG	nodose ganglion
NGF	nerve growth factor
NOD	non-obese diabetic
PI3K	phosphatidylinositol-3-kinase
PKC	protein kinase C
PSN	primary sensory neuron
SNIRKO	sensory neuron insulin receptor knock out
SP	substance P
TrkA	tropomyosin kinase A
TRPV1	transient receptor potential vanilloid type 1
